# A case of giant necrotic spermatic cord lipoma found incidentally during recurrent inguinal hernia repair: A case report

**DOI:** 10.1016/j.ijscr.2022.107760

**Published:** 2022-10-22

**Authors:** Alona Salita, Mohamed Hussein, Qazi Azher, Gul Sachwani-Daswani, Kristoffer Wong

**Affiliations:** a Department of General Surgery, Beaumont Health Farmington Hills, MI, USA; b Division of Trauma Surgery, Department of General Surgery, Hurley Medical Center Flint, MI, USA; c Department of Pathology, Hurley Medical Center Flint, MI, USA

**Keywords:** Case report, Spermatic cord, Lipoma, Giant, Necrotic, Recurrent inguinal hernia

## Abstract

**Introduction and importance:**

The literature regarding size descriptions for spermatic cord lipomas is limited. The term “giant” is utilized loosely and seen on case reports for masses as small as 6 cm. Here we present a case of a giant left sided spermatic cord lipoma, found incidentally during a recurrent inguinal hernia repair, that measured 18 × 14 × 10 cm on final pathologic examination.

**Case presentation:**

A 59 year old male, with a history of morbid obesity and surgical history of prior bilateral inguinal hernia repair, presented with recurrent left sided groin and scrotal bulging with associated discomfort several months after the initial hernia repair. Following imaging performed on preoperative work up, the patient was brought to the operating room for a robotic inguinal hernia repair.

**Clinical discussion:**

Preoperative physical examination was limited due to the patient's body habitus which precluded a definitive diagnosis of inguinal hernia based on physical examination. Computed tomography of the abdomen and pelvis was performed with findings consistent with bilateral recurrent inguinal hernias. A bilateral robotic inguinal hernia repair was attempted. The procedure was converted to open via a groin incision when an incidental 18 cm left sided spermatic cord lipoma was discovered. An orchiectomy was ultimately performed as the mass was intimately intertwined with the spermatic cord.

**Conclusion:**

In our case, the patient had a recurrent inguinal hernia and an incidental finding of an 18 cm spermatic cord lipoma which warranted a left orchiectomy followed by open inguinal hernia repair. We propose standardizing the term “giant” to include spermatic cord lipomas >15 cm.

## Highlights

• Elevated BMI is associated with high rate of hernia recurrence.
• Adequate exclusion of cord lipoma during index hernia repair is necessary.
• Describing benign spermatic cord masses as “giant” should be reserved for masses > 15 cm.

## 1 Introduction

Spermatic cord lipomas are typically found incidentally during inguinal hernia repair procedures. Patients with inguinal hernias typically present with a bulge in their groin and possibly scrotal region. Clinically, it is difficult to differentiate herniating intraabdominal contents in an inguinal hernia from the presence of a spermatic cord lipoma. During inguinal hernia repair procedures, cord lipomas have been discovered between 20 and 70 % of the time. Imaging modalities such as ultrasound, computed tomography and magnetic resonance imaging have been utilized to aid in their diagnosis [1].

According to Priemer et al., in a 17-year retrospective review of paratesticular soft tissue masses, lipomas were the most commonly found benign neoplasm of the spermatic cord. Their data showed that one third of orchiectomies performed for paratesticular masses were pathologically consistent with benign lesions [2]. One case report describes findings of a 10 × 7.5 × 5 cm mass pathologically consistent with a spermatic cord lipoma misdiagnosed as an inguinal hernia [3]. Another report describes a “giant” spermatic cord lipoma, discovered during an inguinal hernia repair, measuring slightly over 6 cm with histology consistent with benign adipose tissue [4]. Several other cases have been described regarding findings of large spermatic cord lipomas [5]. Vashu et al. suggested that “large” spermatic cord lipomas be described for any lipoma >10 cm [6]. Here we present a case of a giant necrotic spermatic cord lipomatous mass found incidentally during a robotic repair of recurrent bilateral inguinal hernia. The repair occurred at a 457 bed community-based, university-affiliated medical center. This case has been reported in line with the SCARE 2020 criteria [7].

## 2 Case presentation

This is a 59 year old male who presented with recurrent bilateral inguinal hernias. He had a medical history significant for morbid obesity (BMI > 45). He had no contributory family history. He was a non smoker and denied routine ethanol use. He underwent bilateral inguinal hernia repair, robotically with mesh, six months prior.

**Fig. 1.1:**
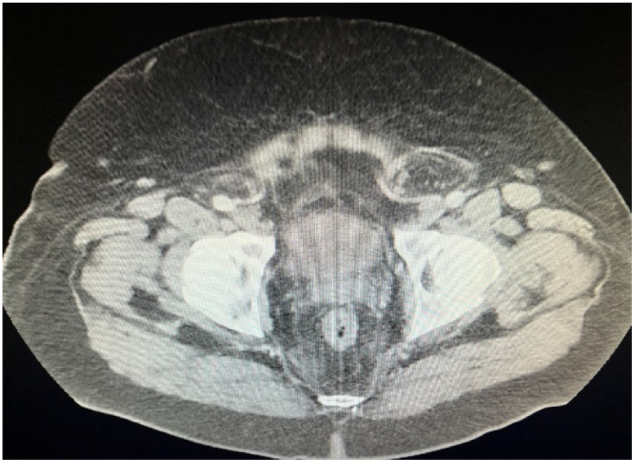
Axial image CT scan revealing recurrent bilateral fat filled inguinal hernias.

**Fig. 1.2:**
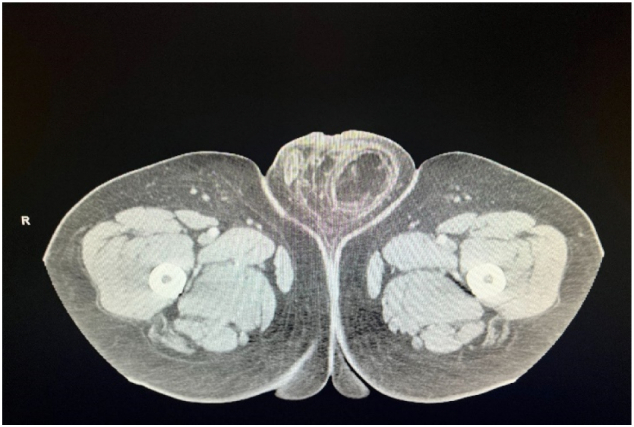
Axial image CT scan with what appears to be a large left fat filled hemiscrotum.

**Fig. 1.3:**
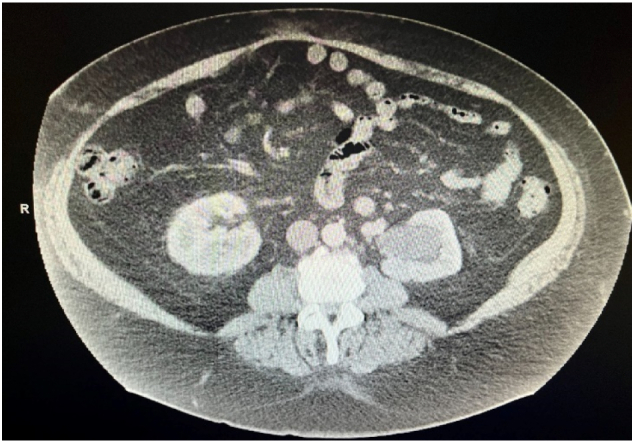
Axial image CT scan demonstrating left hydroureteronephrosis.

Several months after his initial repair, he began complaining of bilateral scrotal swelling, discomfort, and a hard bulge in his proximal left scrotum. CT imaging revealed fat containing bilateral inguinal hernia (Fig. 1.1), left greater than right (Fig. 1.2), with associated left hydroureteronephrosis (Fig. 1.3).

He was taken to the operating room for robotic bilateral inguinal hernia repairs. The repair was performed by the director of the Hernia Center for Excellence who was assisted by an experienced consultant general surgeon. His prior meshes were noted to have migrated superiorly and he had visible recurrent bilateral inguinal hernias. A dissection was performed, including explantation of prior meshes, however the hard “bulge” in the left hemiscrotum was not able to be reduced. Due to concern for a pathologic mass in that area the robotic procedure was terminated and an open inguinal hernia repair was pursued. During the open dissection a large lipomatous mass was reduced out of the left inguinal canal.

**Fig. 2:**
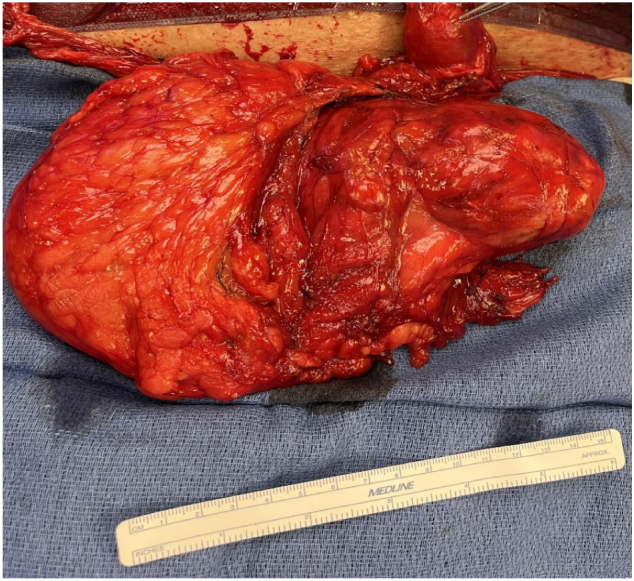
Large lipomatous mass on spermatic cord. Left side of mass with soft normal feeling fatty tissue and right was a hard and encapsulated structure. Top right corner instrument pointing to left testicle.

On tactile analysis, the large mass had a softer more normal fatty texture as well as a harder mass with a capsule associated with it (Fig. 2). The mass was intimately associated with the spermatic cord near the left testicle making dissection of the spermatic cord structures very difficult therefore an en bloc orchiectomy was performed.

**Fig. 3.1:**
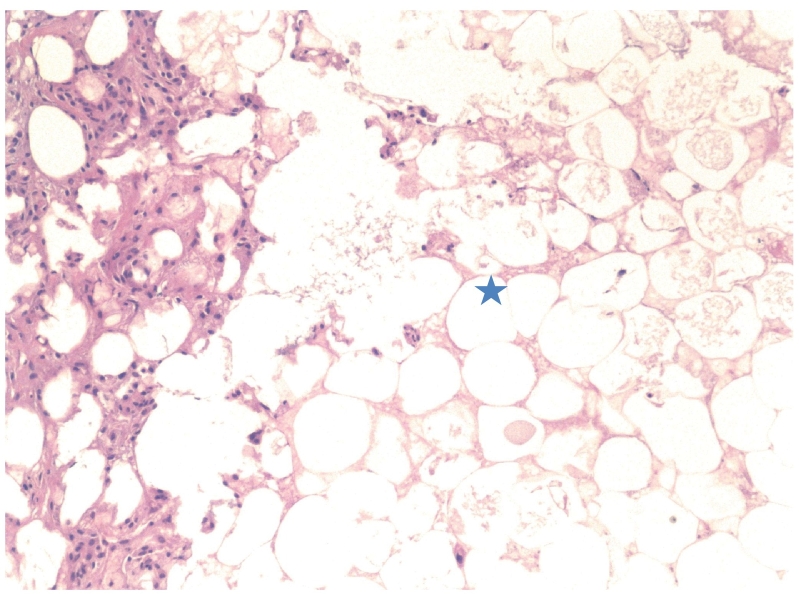
Hematoxylin-eosin stain of fibrofatty tissue of inguinal mass showing an area of fat necrosis (blue star) (original magnification ×100).

**Fig. 3.2:**
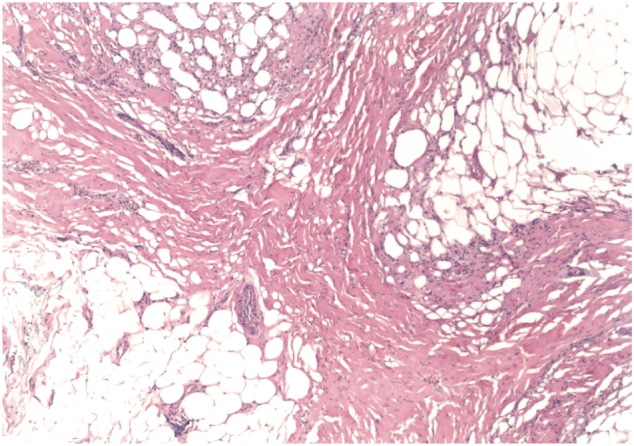
Hematoxylin-eosin stain of left cord mass showing dense fibrosis with adjacent fat necrosis (original magnification ×40).

Final specifications by the pathology department at our institution characterized the mass as encapsulated yellow fibrofatty tissue measuring 18 × 14 × 10 cm with histologic characteristics of non malignant adipose tissue with evidence of fat necrosis (Fig. 3.1, Fig. 3.2). Attached to the mass was a convoluted spermatic cord measuring 21 cm and a left teste, histologically normal, measuring 3.7 × 3.3 × 2.7 cm. The patient recovered well post operatively and was discharged the next day requiring minimal pain control.

## 3 Discussion

Spermatic cord lipomas are usually an incidental finding and are frequently misdiagnosed as inguinal hernias. In many cases, they can present concomitantly. Physical examination alone can't definitively differentiate between both conditions. This can be further complicated in patients with an increased body habitus, as in our patient [1]. In a study of 1194 patients, Schjøth-Iversen et al. found that risk of hernia recurrence was elevated three-fold in patients with BMI >30 when compared to patients with BMI < 30 [8]. Increased oxidative stress and immunosuppressive factors associated with obesity are the likely culprits that preclude proper integration of mesh with surrounding tissue [9]. Therefore, we suggest taking necessary steps to prevent recurrence which include: ensuring adequate mesh coverage, fixation of the mesh to the pubic tubercle and the rectus, and using a larger mesh size that covers femoral, obturator, and both direct and indirect hernias.

Pre operatively, the patient was diagnosed with a recurrent inguinal hernia as the patient had initial hernia repair 6 months prior in a robotic laparoscopic setting. On physical examination, the patient had a groin bulge as well as CT scan findings consistent with recurrent hernia. Intraoperatively, a recurrent hernia was confirmed but on external examination the ipsilateral groin bulge did not resolve with laparoscopic hernia sac reduction. Only upon converting to an open repair were we able to visualize a giant spermatic cord lipoma. Prior literature suggests that in order to avoid missing a spermatic cord lipoma diagnosis, external compression of the superficial inguinal ring should be performed along with internal traction of the cord structures in order to reveal a spermatic cord lipoma during laparoscopic inguinal hernia repair [10].

According to Heller et al., post mortem autopsies revealed a 75 % incidence of spermatic cord lipomas in men ranging in age between 24 and 92 years old [11]. A literature review of spermatic cord lipomas described as “giant” revealed that a lipoma measuring 28 cm × 25 cm × 8 cm was described by Cavazzola et al. [12], a 15 cm × 10 cm × 3 cm spermatic cord lipoma was described by Chang et al. [13], a 10 cm × 9 cm × 5 cm mass described by Kaplanoglu et al. [14], and another 10 cm × 7.5 cm × 5 cm mass described by Edelstein [3].

The range for the term "giant" appears to be loose throughout the literature. Due to the descriptive discrepancy in the literature on size for spermatic cord lipomas, we suggest reserving the term “giant” as it relates to this pathology for tumors >15 cm in size. No case reports were found to describe a giant spermatic cord lipoma with associated necrosis. It is our hypothesis that in our case the spermatic cord lipoma was found to be necrotic on histological analysis likely due to its size and prior hernia repair at that site possibly contributing to tenuous blood supply.

## 4 Conclusion

This case highlights the necessity of adequate exclusion of cord lipoma during minimally invasive or open inguinal hernia index repair in patients with elevated BMI in addition to performing necessary measures to prevent recurrence. This case also highlights the inaccuracy in use of terms to describe the size of benign spermatic cord masses. There have been multiple reports of spermatic cord lipomas discovered incidentally during inguinal hernia repair. Masses as small as 6 cm have been previously described as "giant". In light of the above, we suggest both reserving the term "giant" for benign spermatic cord masses that are >15 cm as well as suture mesh fixation in patients with elevated BMI (i.e., BMI > 30).

## Provenance and peer review

Not commissioned, external peer-reviewed.

## Consent

Written informed consent was obtained from the patient for publication of this case report and accompanying images. A copy of the written consent is available for review by the Editor-in-Chief of this journal on request.

## Ethical approval

Not applicable.

## Funding

None.

## Guarantor

Kristoffer Wong, DO, FACOS.

## Research registration number

Not applicable.

## CRediT authorship contribution statement

Alona Salita (Conceptualization; Data curation; Writing - Original draft; Writing - Review & editing), Mohamed Hussein (Writing - Review & editing), Qazi Azher (Investigation), Gul Sachwani (Conceptualization; Investigation; Data curation; Writing - Original draft; Writing - Review & editing), Kristoffer Wong (Conceptualization; Investigation; Data Curation; Writing - Original draft; Writing - Review & editing).

## Declaration of competing interest

The authors declare that they have no conflicts of interest.
